# Natural Products as Modulators of Nrf2 Signaling Pathway in Neuroprotection

**DOI:** 10.3390/ijms24043748

**Published:** 2023-02-13

**Authors:** Ignacio Moratilla-Rivera, Marta Sánchez, Jose Antonio Valdés-González, María Pilar Gómez-Serranillos

**Affiliations:** Departamento de Farmacología, Farmacognosia y Botánica, Facultad de Farmacia, Universidad Complutense de Madrid, Plaza Ramón y Cajal s/n, Ciudad Universitaria, 28040 Madrid, Spain

**Keywords:** Nrf2, phenolic compounds, terpenoids, oxidative stress, neurodegeneration

## Abstract

Neurodegenerative diseases (NDs) affect the West due to the increase in life expectancy. Nervous cells accumulate oxidative damage, which is one of the factors that triggers and accelerates neurodegeneration. However, cells have mechanisms that scavenge reactive oxygen species (ROS) and alleviate oxidative stress (OS). Many of these endogenous antioxidant systems are regulated at the gene expression level by the transcription factor Nrf2 (nuclear factor erythroid 2-related factor 2). In the presence of prooxidant conditions, Nrf2 translocates to the nucleus and induces the transcription of genes containing ARE (antioxidant response element). In recent years, there has been an increase in the study of the Nrf2 pathway and the natural products that positively regulate it to reduce oxidative damage to the nervous system, both in in vitro models with neurons and microglia subjected to stress factors and in vivo models using mainly murine models. Quercetin, curcumin, anthocyanins, tea polyphenols, and other less studied phenolic compounds such as kaempferol, hesperetin, and icariin can also modulate Nrf2 by regulating several Nrf2 upstream activators. Another group of phytochemical compounds that upregulate this pathway are terpenoids, including monoterpenes (aucubin, catapol), diterpenes (ginkgolides), triterpenes (ginsenosides), and carotenoids (astaxanthin, lycopene). This review aims to update the knowledge on the influence of secondary metabolites of health interest on the activation of the Nrf2 pathway and their potential as treatments for NDs.

## 1. Introduction

Neurodegenerative diseases (NDs) are a diverse group of pathologies characterized by a gradual loss in neuron number and function. These pathologies are primarily caused by the accumulation of misfolded proteins, as seen in Alzheimer’s disease (AD), Parkinson’s disease (PD), and Huntington’s disease, and are associated with a decline in cognitive abilities and movement disorders [1]. In 2019, the estimated number of individuals affected by dementia was 55 million, and it is projected to increase to 152 million by 2050, with a disproportionate impact in developing countries [2]. Moreover, the prevalence of PD has doubled since 1997, with the World Health Organization estimating 8.5 million cases in 2019 [3]. The rapid rise in NDs and their significant economic impact justify the need to find solutions.

The predominant risk factor for neurodegeneration is advanced age, although other factors such as high blood pressure, depression, low educational level, sedentary lifestyle, and oxidative stress (OS) also contribute [2,4,5]. The nervous system is highly sensitive to reactive oxygen species (ROS) which primarily originate in the mitochondria but can also come from external sources. When ROS exceed the neutralization systems of the cell, OS is generated [6]. The organism’s antioxidant capacity is dependent on the presence of enzymes that break down ROS such as superoxide dismutase-1 (SOD-1), heme oxygenase-1 (HO-1), NADPH (nicotinamide adenine dinucleotide phosphate) quinone reductase-1 (NQO-1), catalase (CAT), and peroxidase, as well as internal free radical scavengers such as NADPH, glutathione (GSH), and coenzyme Q, and external such as vitamins A, C, and E [7,8,9]. The ability to modulate antioxidant mechanisms could be a useful tool to mitigate nervous damage associated with OS.

Phytochemicals are associated with an improvement in human health and lifespan due to their antioxidant properties, which decrease OS and reduce the toxicity of diseases such as cancer, cardiovascular diseases, and NDs [10,11]. While several studies support the benefits of natural products such as curcumin, resveratrol, epigallocatechin gallate, and quercetin on NDs, further research is needed to use them in the treatment of conditions such as AD or PD [12]. In recent years, there has been growing interest in studying the Nrf2 (nuclear factor erythroid 2-related factor 2) pathway, both in basic research and in clinical applications. The focus is especially on natural compounds that could modulate the activity of the pathway and thus alleviate OS [13].

The purpose of this review is to present the current knowledge of how various natural compounds can impact the Nrf2/ARE (antioxidant response element), pathway and to explore the potential for using this pathway as a target for treating NDs. The review included in vitro and in vivo studies of the interaction of natural compounds with the Nrf2 pathway and their properties to alleviate OS and neuronal damage. The search of the literature was elaborate, using a combination of the following keywords “Nrf2”, “neurodegeneration”, “phenolic compounds”, “terpenoids”, “ginkgolides”, “gingenosides”, “quercetin”, “curcumin”, “resveratrol”, “hydroxytyrosol”, “monoterpenes”, and “flavonoids” on PubMed, Google Scholar, Wiley, ScienceDirect, Springer, and Cochrane. The articles collected are from 2018 to 2022 and written in English.

## 2. Nrf2 Signaling Pathway against OS

The transcription factor Nrf2 is a critical cellular protein involved in combating OS and eliminating xenobiotics from the organism [14]. This transcription factor belongs to the “cap’n’collar” subfamily (CNC) with basic leucine zipper (bZIP) that allows it to bind to DNA (deoxyribonucleic acid) [15]. It is a modular protein composed of seven domains known as Neh 1-7 (Nrf-ECH homology domain) [16]. Nrf2 has been linked to resistance to infections, tumor resistance to chemotherapeutics, and, as this review explores, protection against NDs [17,18,19].

Nrf2 is negatively regulated by Keap-1 (Kelch-like ECH-associated protein 1) under normal conditions. Keap-1 binds to the DLG (Asp-Leu-Gly) and ETGE (Glu-Thr-Gly-Glu) motifs of the Neh2 domain and recruits the Cul3 (cullin3)/Rbx1 (RINGBox1) E3 ubiquitin ligase complex, which leads to the ubiquitination of lysine residues of Nrf2 and subsequent degradation in the proteosome [20,21]. However, when cells are subjected to OS, the cysteine residues of Keap-1 become oxidized and release Nrf2, increasing its intracellular levels. Free Nrf2 then penetrates the nucleus, binds to sMaf (small muscle aponeurosis fibromatosis), and couples to cis-regulatory elements of certain genes called ARE specifically to the sequence 5′-TGACXXXGC-3′ [16,22]. The expression of enzymes responsible for ROS scavenging is regulated by the Nrf2-sMaf heterodimer, such as HO-1, SOD, NQO-1, and glutathione peroxidase (GPx), among others [14,23,24].

**Figure 1:**
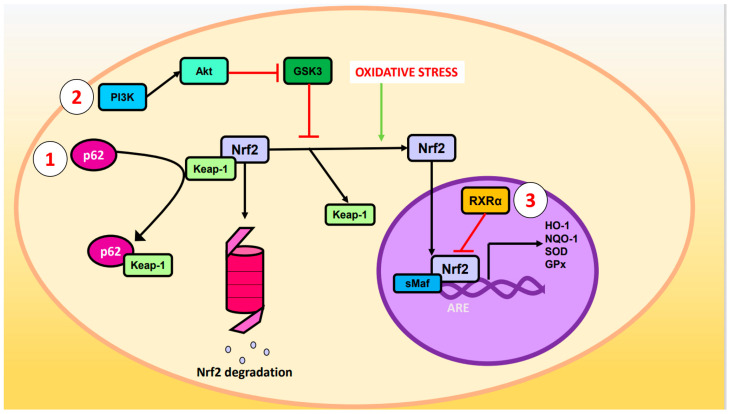
Regulation of Nrf2. Oxidative stress causes cleavage of Nrf2 and Keap-1 such that Nrf2 ceases to be degraded in the proteasome and enters the nucleus where it forms a heterodimer with sMaf and induces the expression of ARE genes such as HO-1, NQO-1, SOD, and GPx. The Nrf2 pathway can be modulated by the (1) p62, (2) PI3K/Akt/GSK-3, or (3) RXRα pathways.

Other ways by which the pathway is regulated are illustrated in Figure 1:

1. p62 phosphorylated at Ser-351: p62, also known as sequestosome 1, is a multifunctional protein involved in various cellular processes, including autophagy and OS response. Upon phosphorylation, p62 acquires high affinity for Keap-1, preventing Nrf2 ubiquitination and degradation [25].
2. Glycogen synthase kinase-3 (GSK-3): GSK-3 is a Ser/Thr kinase that negatively regulates Nrf2 by phosphorylating Ser residues of the Neh6 domain. The phosphorylated residues are recognized by the E3 adaptor ligase β-TrCP (β-transducin repeat-containing protein), which recruits the Cul3/Rbx complex, ubiquitinates the Nrf2, and leads to its degradation. The PI3K (phosphatidylinositol-3 kinase)/Akt (protein kinase B) pathway can inhibit GSK-3 and prevents Nrf2 phosphorylation. Likewise, PI3K/Akt can be activated by ion channels, growth factors, and G coupled-protein receptor ligands [26].
3. RXRα (retinoid X receptor α): this transcription factor associates with the Neh7 domain of Nrf2 to block the expression of genes related to decreased OS [27].

## 3. Phenolic Compounds

Phenolic compounds are a group of plant phytochemicals with an aromatic ring linked to hydroxyl groups. These natural compounds are attributed with several benefits on human health such as the prevention of diabetes, cardiovascular diseases, and NDs. They work through antioxidant and anti-inflammatory properties, sometimes by modulating Nrf2 or NF-κB (nuclear factor kappa-light-chain-enhancer of activated B cells) signaling pathways [28]. The findings are succinctly presented in Table 1 for the in vitro models and Table 2 for the corresponding in vivo models, arranged in the order of their occurrence in the manuscript.

### 3.1. Quercetin

Quercetin is a flavonol found in fruits, vegetables, and tea. It has been studied for its effects on the Nrf2 pathway in in vitro and in vivo models. A methanolic extract from the dried leaves of *Dendropanax morbifera* was used to isolate and characterize quercetin and isoquercetin which showed a protection against glutamate-induced ROS production in HT22 cells. When the cells were treated with 4 mM glutamate, Nrf2 and HO-1 expression increased, and the addition of isoquercetin (50 and 100 μM), quercetin (5 and 10 μM), or the extract of *D. morbifera* led to a higher induction of expression, indicating a protective effect of these flavonoids against Glu-generated toxicity [29]. Cholesterol oxidation forms 7-ketocholestrol (7KC) which causes oxidative damage in neurons. In vitro, N2a cells were cultured with 7KC (50 μM for 48 h), and adding polyphenols (apigenin, resveratrol, or quercetin) diminished the cytotoxic effects. All three compounds studied (3.125 μM) increased Nrf2 and SOD-1 mRNA (messenger ribonucleic acid) levels compared to those treated with 7KC alone, but quercetin had the greatest increase in the protein levels of Nrf2, SOD-1, SOD-2, GPx, and CAT over resveratrol and apigenin. However, SOD and GPx activities did not differ significantly between the different polyphenols [30]. Corticosterone (200 μM for 96 h) was used as a stressor on primary rat cerebral cortex cells (neurons and astrocytes) causing morphological changes and apoptosis. Pretreatment with quercetin (3 μM for 24 h) increased viability but did not seem to be mediated by Nrf2 action as the genes regulated by this factor such as HO-1, NQO-1, and glutamate–cysteine ligase catalytic subunit (GCLC) did not show a significant increase in expression. Nrf2 inhibition with trigonelline did not affect the neuroprotective action of quercetin. The decrease in corticosterone-induced cell death was proposed to be due to the increased levels of FKBP5 (FK509 binding protein 5, a negative regulator of the glucocorticoid receptor) produced by quercetin [31]. In ΔK280 TauRD SH-SY5Y cells, treatment with quercetin (5 μM for eight hours) increased Nrf2 levels and reduced OS caused by ΔK280 TauRD. Similar results were seen with apigenin and 7,8-dihydroxyflavone (7,8-DHF). Chiang et al. (2021) proposed that the activation of the Nrf2 pathway may be partly mediated by the activation of tropomyosin receptor kinase B (TRKB), a brain-derived neurotrophic factor (BDNF) receptor, which upregulates Akt activation, an activator of Nrf2, and thus decreases the mutant Tau protein aggregation [32]. Another assay in SH-SY5Y cells stressed by high glucose concentration showed that quercetin improved cell viability through an upregulation of glyoxalase 1 activity. The increased expression of this ARE-regulated gene was due to an increase in the amount of Nrf2, p-Nrf2, and their translocation to the nucleus. In addition, inhibitors of Nrf2 regulatory kinases were employed which led to the conclusion that the activation of this factor could be mediated by protein kinase C (PKC) activation and/or inhibition of GSK-3β [33]. An in vitro study was performed using PC12 cells exposed to Aβ_25-35_ (amyloid β) (20 μmol/L for 24 h) to simulate AD. Prior treatment with quercetin (80 μmol/L) was found to enhance cell survival and increase antioxidant enzyme activity (SOD, CAT, and GPx). Despite these positive effects, pretreatment with quercetin resulted in decreased levels of Nrf2 and its positive regulator Sirtuin-1 (SIRT1) mRNA and protein, which did not occur with HO-1 [34].

Glioma C6 cells exposed to acrylamide were found to exhibit similar behavior as observed in previous studies. Results obtained through immunocytochemical analysis revealed that treatment with acrylamide led to an increase in Nrf2 levels, and pretreatment with quercetin (2 μM) followed by acrylamide (4 μM) resulted in a further increase in Nrf2 levels with grater nuclear staining than cytoplasmatic staining. Thymoquinone also displayed similar effects, although a higher concentration (3.9 μM) was required [35].

An in vivo study was performed using Sprague Dawley (SD) rats intracranially injected with Aβ_42_ for 15 days to establish a model of AD. For the next 18 days, one of the groups of rats received daily oral administration of quercetin (100 mg/kg) dissolved in olive oil, which exhibited improved memory, reduced accumulation of Aβ aggregates, and lower OS levels. Nrf2 levels were increased by quercetin treatment, in addition to antioxidant proteins (CAT, SOD, HO-1, and GSH). These effects were more pronounced when quercetin was combined with a DPP4 (dipeptidyl-peptidase 4) inhibitor (sitagliptin) [55]. Streptozotocin (3 mg/kg for three days) given intracerebroventricularly in Wistar rats was used as a model of AD. Rats co-treated with quercetin (50 mg/kg for 18 days) had improved memory and cholinergic dysfunction and decreased Aβ aggregates compared to those treated with streptozotocin alone. Additionally, quercetin showed an increment in α7nAChR (α7 nicotinic acetylcholine receptor) and HO-1 levels. The effects presented by quercetin were reversed by the coadministration of trigonelline (Nrf2 inhibitor) and methyllycaconitine (α7nAChR inhibitor), so a possible α7nAChR/Nrf2/HO-1 signaling pathway activated by quercetin was suggested [56]. The action of quercetin (50 mg/kg/day) to palliate the neurotoxic effects generated by an energy drink (7.5 mL/day) was also studied in Wistar rats. In the group receiving the beverage, an increased release of proinflammatory molecules (IL (interleukin)-1β) and increased OS were experienced. In the group receiving the beverage + quercetin, a decrease in IL-1β and OS was observed, possibly due to augmented transcription and expression of Nrf2 and HO-1 [57]. The same deoxidant and anti-inflammatory response was reported in SD rats with traumatic brain injury (TBI) that received quercetin treatment at the same dose as in the previous experiment. An increase in the protein levels of Nrf2 (in both the nucleus and cytoplasm) and HO-1 was also perceived, in addition to increased CAT, SOD, and GPx activities [58]. Kunming mice subjected to chronic unpredictable mild stress experienced inflammatory and oxidative damage in the hippocampus, in addition to decreased amounts of SOD, GST (glutathione-S-transferase), p-PI3K, p-Akt, Nrf2, and HO-1. Quercetin-treated mice (40 mg/kg/day) experienced an increased expression of the proteins above. The proposed pathway through which quercetin works was PI3K/Akt/Nrf2/HO-1 [59]. In another experiment, Wistar rats were subjected to transient middle cerebral artery occlusion (tMCAO), and the group that received quercetin treatment showed a relief in infarct volume, neuronal impairments, and blood–brain barrier permeability. Quercetin elicited the expression of SIRT1, Nrf2, and HO-1, so the neuroprotective activity was suggested to be mediated by the SIRT1/Nrf2/HO-1 pathway. To test this, a SIRT1 inhibitor (EX527) blocked quercetin protection [60].

### 3.2. Curcumin

Curcumin, a phenolic compound present in *Curcuma longa* rhizome, has anti-inflammatory and antioxidant properties. The activation of Nrf2 by curcumin is described in in vitro experiments such as the one performed by Park et al. (2021) in mouse cerebral cortex cells. They demonstrated that cells treated with curcumin (10 μM) had higher expression of Nrf2, NQO-1, GST, and HO-1, as well as exhibiting Nrf2 translocation to the nucleus and the transcriptional activity of ARE genes. The proposed mechanism for this regulation involves the PKCδ activation by curcumin, which phosphorylates p62 at Ser-351, and this prevents the inhibition of Nrf2 by Keap-1 [36]. By providing lower concentrations of curcumin (2 μM) to HT22 cells stressed with ethanol (100 μM), an increase in Nrf2 and HO-1 expression was demonstrated with respect to the ethanol-treated cells. The effect was reversed when Nrf2 was silenced by siRNA (small interfering RNA) [37].

In a study of primary cultures of SD rat astrocytes exposed to methyl-mercury (5 μM for 6 h), pretreatment with curcumin showed a decrease in ROS and an increase in CAT activity, GSH content, Nrf2 (both cytoplasmic and nuclear), HO-1, and NQO-1. However, the effect of curcumin was reversed when Nrf2 siRNA was used. The transduction pathway was tested by PKCδ inhibitors, concluding that curcumin protects against methylmercury independently of this kinase [38].

Microglia studies (HAPI cells and primary cortical microglia culture) attempted to recreate the prooxidative conditions of intracranial hemorrhage by adding heme (20 μM) to the medium. When treatment with heme + curcumin (10 μM) was added, the amount of ROS decreased, but this did not occur in the presence of ML385, an Nrf2 inhibitor. Moreover, these results were complemented by others in which an increase in the transcription and protein amount of Nrf2, HO-1, NQO-1, and GPx4 was observed [39]. Experiments performed on Pam3CSK4-stimulated BV2 microglia revealed that curcumin increased HO-1 expression in a dose-dependent manner, with the maximum induction observed at eight hours with 20 μM treatment. Furthermore, curcumin treatment was found to increase translocation of Nrf2 to the nucleus at two hours. Additionally, the role of curcuminoid in inhibiting pathways associated with NF-κB and p38MAPK (mitogen-activated protein kinase) inflammation was determined [40]. The potential of curcumin analogues and derivatives that show greater stability and ability to activate Nrf2 has also been investigated [70,71].

In an in vivo model of diffuse axonal injury in SD rats, pretreatment with curcumin (20 mg/kg) decreased neurodegeneration and incremented Nrf2 translocation to the nucleus. The transduction pathway was further investigated, and it was concluded that curcumin induces phosphorylation of p-ERK (extracellular-signal-regulated kinase), and this promotes the mobilization of Nrf2 to its target genes [72]. The neuroprotective potential of curcumin was analyzed in a TBI model in ICR mice, and it was shown that this compound scavenged ROS, raised SOD and GPx activities, and provoked Nrf2 translocation to the nucleus where it promotes gene expression (HO-1 and NQO-1) [61]. Another TBI model was performed in Nrf2 knockout mice, in which the protective effect of curcumin was lower compared to wild-type (WT) mice [62]. OS produced by chronic unpredictable mild stress in SD rats was attenuated by daily treatment with curcumin (100 mg/kg for 28 days) which upregulated Nrf2, HO-1, and NQO-1 transcription [63]. Curcumin (400 mg/kg/day for six days) was able to mitigate nerve damage caused by quinolinic acid in Wistar rats. This was demonstrated to be related to the Nrf2 pathway as it increased Nrf2 levels and the levels of genes regulated by it (SOD, CAT, GST, GSH, GPx). It was proposed that curcumin elevates the levels of BDNF, a ligand of TRKB, which activates ERK1/2 and this in turn activates Nrf2, resulting in the expression of genes that protect against neuronal damage [64]. Finally, research has been conducted in non-mammalian in vivo models. Sanshui white ducks were subjected to arsenic-trioxide-induced neurotoxicity, resulting in a decrease in the expression of Nrf2 and the genes regulated by it (HO-1, thioredoxin, GPx, SOD-1, CAT), and an increase in Keap-1 levels. Dietary supplementation with curcumin had the opposite effects, demonstrating its neuroprotective action through the Nrf2 pathway [65].

### 3.3. Other Phenolic Compounds

#### 3.3.1. Phenolic Compounds of Olive Tree

Hydroxytyrosol is a biologically active compound found in the fruits and leaves of the olive tree (*Olea europaea*) and is known for its potent antioxidant properties, making it a potential candidate for addressing NDs. Isolated hydroxytyrosol (5 and 10 μM) produced non-significant variations in the amount of Nrf2 and did not neutralize the appearance of ROS generated by H_2_O_2_ in SH-SY5Y cells [41]. With these results, it can be hypothesized that the neuroprotective effect of olive oil is due to a synergistic action of its phenolic composition. The antioxidant activity in SK-N-SH cells treated with H_2_O_2_ of a mixture of olive oil phenols (oleuropein, *p*-coumaric acid, and tyrosol) was studied. The result showed that the compendium of the three substances improved cell viability and decreased ROS. However, Nrf2 levels decreased when the mixture was applied [42].

An experiment was conducted using an olive dry extract enriched with 20% hydroxytyrosol on *Caenorhabditis elegans*. The extract was found to decrease the aggregation of Aβ and tau proteins in *C. elegans* mutants. Furthermore, the study demonstrated an increase in the nuclear translocation of skinhead-1 (a transcription factor homologous to mammalian Nrf2) resulting in elevated expression [66].

#### 3.3.2. Anthocyanins and Proanthocyanidins

In an in vitro AD model with HT22 cells exposed to AβO (amyloid β oligomer), it was found that anthocyanins (100 μg/mL) improved cell viability and decreased cytotoxicity, but when combined with PI3K and Nrf2 inhibitors these results were not observed [43]. Another experiment performed in HT22 cells evidenced that pretreatment with cyanidin-3-glucoside (C3G) reduced ROS and glutamate-induced cytotoxicity. It was proposed that C3G raised Nrf2 levels by ERK activation, as the levels of both increased in a dose-dependent manner. Moreover, there was an upregulation in the transcription of deoxidative enzymes (SOD-1, SOD-2, GPx, and CAT) [44]. On the other hand, the treatment of mouse cortical neurons with proanthocyanidins, a type of flavanol oligomer, reversed the detrimental effects caused by cypermethrin. However, there was a reduction in Nrf2, HO-1, and NQO-1 expression. The authors explained this as a homeostatic response, as an excessive activation of antioxidant systems can also lead to cellular damage [45].

Korean black bean anthocyanins purified from methanolic extract were studied in murine AD models. Anthocyanin treatment was administered at a dose of 12 mg/kg for 30 days in both WT mice and APP (amyloid-β precursor protein)/PS1 (presenilin-1) double mutants. The results obtained evidenced the capacity of these molecules to increase HO-1, glutamate–cysteine ligase modifier subunit (GCLM), and Nrf2 levels, resulting in an alleviation of OS. Likewise, it was determined that this Nrf2 activation was mediated by the PI3K/Akt/GSK-3β pathway [43].

#### 3.3.3. Flavonoids

The flavonoid concept encompasses a set of secondary metabolites of phenolic nature that have a multitude of biological activities that have a positive impact on human health [73]. Apart from quercetin, studies have been conducted on other isolated flavonoids, although not as assiduously. The neuroprotective capacity of kaempferol was tested in a culture of primary mouse cortical neurons subjected to nutritional stress. Neurons treated with kaempferol (10 μM) showed less oxidative damage and higher amounts of antioxidant systems such as SOD and GSH. Furthermore, it was demonstrated that it had the capacity to induce the expression of Nrf2, GPx4, and Solute Carrier Family 7 Member 1 (SLC7A1), a cysteine–glutamate antiporter. However, when an Nrf2 inhibitor was added, the effect was reversed. It was proposed in this study that the kaempferol-activated Nrf2/SLC7A1/GP×4 pathway might be responsible for its protective action [46].

Tiliroside, a kaempferol-containing glycoside, increased the levels of nuclear Nrf2, HO-1, and NQO-1 in both HT22 cells and BV2 microglia at concentrations between 4 and 6 μM, thus allowing a higher antioxidant capacity of the cell types [47]. Another experiment performed on BV2 cells stimulated by Aβ_1-42_ indicated that engeletin (dihydrokaempferol 3-rhamnoside) at 20 and 40 μM concentrations increased cell viability, improved the antioxidant capacity of the cells, decreased the release of proinflammatory cytokines, and manifested a raise in the amount of Nrf2 and a downregulation of Keap-1 in a dose-dependent manner. The silencing of Nrf2 by siRNA overrode the above results, demonstrating that engeletin protection occurs through the activation of this transcription factor [48]. A glycoside of kaempferol, icariin (40 μM), decreased the toxicity produced by 6-OHDA (6-hydroxydopamine) in co-cultures of mouse neurons and microglia. Furthermore, it became clear that the effect occurred due to the activation of the Nrf2 pathway in microglia and not in neurons. The upregulation of icariin on Nrf2 was also demonstrated in murine models of PD, as the neuroprotection against 6-OHDA observed in WT mice was not present in Nrf2 knockout mice [49]. 

Isoliquiritigenin is a flavonoid present in licorice root. This compound at 10 and 20 μM concentrations demonstrated anti-inflammatory and antioxidant activity in microglia BV2 cells stimulated by AβO, as it increased the activation of the Nrf2/HO-1 pathway and inhibited NF-κB. Consequently, nitric oxide production and proinflammatory cytokines that generate neuronal damage in AD were reduced [50].

A subgroup within the flavonoids are the flavanones, among which are hesperetin and pinocembrin. In vitro studies demonstrated the neuroprotective effects of pinocembrin-7-methyleter (PME) against 6-OHDA-induced neurotoxicity in SH-SY5Y cells. The results showed that PME improved cell viability, reduced apoptosis, and enhanced antioxidant activity in a dose-dependent manner. The findings indicated that PME decreased cytoplasmic Nrf2 but increased nuclear Nrf2, activating the ARE promoter and increasing the expression of HO-1 and NQO-1. Nrf2 silencing (siRNA) abolished PME-mediated protection, highlighting its role in modulating the Nrf2/HO-1 pathway. Mechanistically, PME-induced positive regulation of Nrf2 was found to occur in part through phosphorylation of Akt and ERK [51]. To recreate a model of AD, Aβ_1-42_ was injected into the brains of mice. In one of the experiment groups, Aβ_1-42_ and hesperetin (50 mg/kg/day for six weeks) were simultaneously injected, and this group presented better memory than mice treated only with Aβ_1-42_. The mechanisms through which hesperetin acted on the cerebral cortex and hippocampus were a decrease in OS by the activation of Nrf2/HO-1, the alleviation of neuroinflammation by the downregulation of TLR4 (Toll-like receptor 4)/NF-κB, and a reduction in the intensity of apoptosis [67]. Another similar experiment showed the same results but using LPS (lipopolysaccharide) as a stressor [74]. Based on this evidence, the protective action of hesperetin through the Nrf2/ARE pathway is evident.

*Abelmoschus esculentus* is a plant employed in traditional Chinese medicine, and it contains a wide variety of bioactive flavonoids. The beneficial effects of *A. esculentus* flower ethanolic extract with high flavonoid content (788.56 mg/g) on OS were studied in transient cerebral ischemia–reperfusion injury (TCIRI). For this purpose, in an in vivo model of TCIRI, Kunming mice were treated with different concentrations of the extract. It was observed that the extract was able to decrease oxidative damage by scavenging ROS and modulating the Nrf2/HO-1 pathway [68].

#### 3.3.4. Tea Polyphenols

Tea is a widely consumed beverage globally, offering a diverse range of phenolic compounds that have been reported to have pharmacological activities that enhance human health, including the prevention of cancer, cardiovascular diseases, diabetes, and NDs [75]. Six tea polyphenols (garlic acid (GA), epigallocatechin (EGC), epicatechin-3-gallate (EGG), epigallocatechin-3-gallate (EGCG), theaflavin (TF), and tannic acid (TA)) were analyzed for their capacity to combat dopamine-induced neurotoxicity characteristic of PD. It was found that the higher the amount of hydroxyl groups and aromatic rings, the greater the protection against damage caused by oxidized dopamine in vitro. Additionally, only GA, EGC, and TA showed the capacity to alleviate OS through Nrf2/ARE. To study the induction of this signaling pathway, plasmids with ARE-luciferase in their sequence, some WT ARE-pGL3 and some ARE-pGL3 mutants, were designed and transfected to SH-SY5Y cells. In cells treated for six hours with GA, EGG, and TA, a significant difference in luciferase activity was reported between WT ARE-pGL3 and ARE-pGL3 mutants. Furthermore, they were found by Western blot to increase HO-1 levels. Thus, GA, EGG, and TA activated the Nrf2 pathway, which did not occur with EGCG, TF, and EGC treatments [52]. However, in experiments in PC12 cells subjected to nutritional stress, it was found that TF isolated from the ethanolic extract of black tea promoted Nrf2 translocation to the nucleus, ARE activation, and elicit HO-1 expression, using lower concentrations compared to the previous experiment [53]. However, it is important to acknowledge that the comparison is between two distinct cell lines, and the type of stressor applied also differed; hence, variations in results are expected. 

Other studies contradict that EGCG does not participate in Nrf2 modulation. The protective action of EGCG was studied in vitro in BV2 cells subjected to CoCl_2_-induced hypoxia. The results showed that EGCG increased cell viability and decreased proinflammatory markers (cyclooxigenase-2, IL-6, and nitric oxide synthase). The underlying mechanism consisted of NF-κB inhibition and Nrf2/HO-1 upregulation [54]. Chronic cerebral hypoperfusion (CCH) generates OS that is palliated by EGCG in in vivo models conducted with SD rats. By Western blot, it was found that the amount of PI3K, p-Akt, Nrf2, and HO-1 increased when CCH rats received EGCG. To confirm that this polyphenol mediated its action through the PI3K/Nrf2/HO-1 pathway, inhibitors were used at different levels of the cascade, and with all of them the effects of EGCG were masked [69]. In [54], a microglial (BV2) cell line was utilized instead of a neuronal cell line (SH-SY5Y), which may explain the divergent observations of the effects of EGCG on Nrf2. However, the positive regulation of EGCG on Nrf2 in the in vivo model reported in [69] further strengthens the evidence for the hypothesis that this compound exerts a neuroprotective effect via this pathway.

## 4. Terpenoids

The results are summarized in Table 3 for the in vitro models and Table 4 for the in vivo models, in the order in which they appear in the text.

### 4.1. Monoterpenes

Aubucin belongs to the iridoid glycoside family. It has demonstrated an anti-inflammatory bioactivity, and it has antioxidative properties too. Aucubin was studied in SH-SY5Y neurons to examine its ability to counteract H_2_O_2_-induced OS. The results show that aucubin scavenged ROS and increased antioxidative enzymes such as SOD, CAT, and GPx. The increase in cellular viability with aucubin comes from the activation of the Nrf2/HO-1 signaling pathway [76]. This regulation is produced by an activation of the Nrf2/ARE signaling pathway. Firstly, aubucin promoted the expression and nuclear translocation of Nrf2; consequently, HO-1 or NQO-1 are increased. Moreover, aubucin reduced ROS generation and increased the expression of deoxidative proteins such as GSH, GPx, and SOD. The studied concentrations were 50 µg/mL, 100 µg/mL, and 200 µg/mL for in vitro experiments and 20 and 40 mg/kg for in vivo experiments [77]. 

Dihydroactinidiolide is a monoterpene lactone from many plants such as black tea, silver vine, and tobacco that shows bioactivity through Nrf2 activation. In a model of OS induced by sodium dithionite, glutamate, Aβ, and colchicine on SH-SY5Y cells, dihydroactinidiolide reduced neuronal damage through diminishing ROS, nitrite content, or lipid peroxidation. Western blot assays revealed that dihydroactinidiolide increased Nrf2 and HO-1 levels at an optimum dose of 270 nM [78].

Paeoniflorin is a monoterpenoid glycoside from *Paeonia lactiflora* with a neuroprotective bioactivity which has demonstrated in vitro its ability to increase the Nrf2 signaling pathway. It was studied with SH-SY5Y cells inducing excitotoxicity with glutamate [79]. 6′-*O*-galloylpaeoniflorin is a paeoniflorin derivative with an interesting antioxidative bioactivity that has been studied as a ROS scavenger. It was demonstrated in vitro and in vivo that it reduces inflammation and OS by promoting the PI3K/Akt/Nrf2 pathway [80]. 

Carveol is a monocyclic monoterpenoid with anti-inflammatory and antioxidant properties. It is found in various natural sources such as black tea, orange peel essential oil, and mandarin. In an epilepsy model induced by pentylenetetrazole treatment in rats, carveol (10 and 20 mg/kg) was found to upregulate the expression of Nrf2, resulting in improved inflammatory profiles through the activation of the Nrf2 antioxidant pathway [97]. In a study, SD rats were treated with LPS to induce depression-like symptoms, and administration of carveol at doses of 20 mg/kg and 50 mg/kg demonstrated its ability to mitigate the LPS-induced effects and decrease OS by modulating the Nrf2 pathway [98].

Catalpol is an iridoid glycoside found in various plant families such as Scrophulariaceae, Bignoniaceae, Plantaginaceae, and Lamiaceae. In a study on an epileptic rat model, catalpol (5 mg/kg) increased SOD activity and Nrf2 expression, suppressed Keap-1 expression, and stimulated ARE compared to the epileptic rats group [99]. Models of stress-induced depression showed that Nfr2 and HO-1 are downregulated, but catalpol (10 mg/kg) was shown to restore their levels and may target depression via the PI3K/Akt/Nrf2/HO-1 signaling pathway [100]. Studies on hyperglycemia-induced depression showed that catalpol (5, 10, and 20 mg/kg) reversed pathological phosphorylation of PI3K and Akt and restored Nrf2 and HO-1 levels, attributed to the upregulation of the PI3K/Akt/Nrf2/HO-1 signaling pathway [101].

### 4.2. Ginkgolides

*Ginkgo biloba*, a tree native to Asia, has been employed for centuries as a medicinal natural remedy. Its phytochemical composition includes ginkgolides, a type of diterpene that has protective activity on the nervous system [118].

There are several assays with isolated ginkgolides. To model cerebral ischemia, SH-SY5Y cells were cultured in an oxygen- and glucose-deprived medium and subjected to oxidative damage due to nutritional stress. The effect and mechanism by which ginkgolides A, B, and K (GA, GB, and GK) counteract OS were studied. Among the three, GB (25 mg/L) showed the most significant increase in SOD activity and decrease in ROS in stressed cells. GB treatment led to an increase in the expression of HO-1, NQO-1, p-Nrf2, and p-Akt. Additionally, p-Nrf2 levels and cell viability decreased when GB was combined with an inhibitor of Akt phosphorylation. It was concluded that GB can induce antioxidant mechanisms through the Akt/Nrf2/ARE pathway [81]. 

The same in vitro model of OGD stress was performed in PC12 cells, and in this case it was seen that GA, GB, and ginkgolide C (GC) at 10 μmol/L can activate Akt, which in turn induces Nrf2 activity and its translocation to the nucleus to elicit the expression of antioxidative enzymes such as HO-1. In addition, the ability of Akt to activate CREB (cAMP-response element binding protein) to induce Bcl-2 (B-cell lymphoma 2) synthesis and thus inhibit apoptosis was also observed in this experiment. The treatment of PC12 cells with diterpene ginkgolides meglumine injection (DGMI), a mixture of GA, GB, GC, and GK, exhibited similar effects as isolated ginkgolides at a concentration of 20 μmol/L [82]. 

Furthermore, GK was shown to prevent demyelination and exert a neuroprotective action in in vivo and in vitro experiments with astrocytes. The proposed underlying mechanism was that GK stimulated the IGF (insulin-like growth factor)/PI3K/Nrf2/ARE signaling pathway and, in turn, Nrf2 inhibited NF-κB. This resulted in an anti-inflammatory and antioxidant response of astrocytes that protects against neurodegeneration [83].

The Xingxiong injection (XI), a traditional Chinese medicine containing Ginkgo leaf extract, is used for the treatment of cerebral ischemia/reperfusion injury. An in vivo study was conducted using SD rats with MCAO to determine the mechanism of action of XI. The results showed that XI reduced apoptosis, OS, and inflammation in the infarcted tissue via activation of the Akt/Nrf2/HO-1 pathway [102]. However, it is important to note that the extract contains not only ginkgolides but also flavonoids and trilactones, which could also contribute to the modulation of Nrf2 [118]. The neuroprotective effects of ginkgolide B (GB), a component of the extract, were investigated using the MCAO model in SD rats [81]. The results were consistent with those observed in the study of XI, suggesting that the neuroprotective action of the ginkgo leaf extract [102] could be partially attributed to GB and the activation of the Akt/Nrf2/HO-1 pathway.

The protective effects of DGMI were also evaluated in SD rats subjected to ischemia/reperfusion injury and demonstrated an improvement in neurological function and a decrease in infarct brain volume. The results suggest that DGMI also activated the Akt/Nrf2/HO-1 pathway, playing a crucial role in ginkgo’s neuroprotective effects [82].

### 4.3. Ginsenosides

*Panax ginseng* root is a natural remedy used in traditional Chinese medicine, and its main active ingredients are ginsenosides, i.e., triterpene saponins reported to have various health benefits such as anti-inflammatory and antioxidant effects [119]. Within the ginsenosides, there are many types depending on the substituents present. In this review, ginsenosides Re, Rg1, Rb1, and Rd are discussed.

Ginsenoside Re: Ginsenoside Re showed neuroprotection in an in vitro model of AD consisting of Aβ_25–35_-induced cytotoxicity in SH-SY5Y cells. Treatment with Re (25 μM) resulted in increased cell viability and decreased apoptosis, and OS was alleviated by the activation of Nrf2 and the deoxidizing enzymes HO-1 and NQO-1. To verify that the effect was mediated through Nrf2, Nrf2 was silenced by siRNA, and the beneficial effects on viability and OS vanished [84]. Another experiment with Re (5 μM), but in a PD model with rotenone-intoxicated SH-SY5Y cells, evidenced the same action on viability, apoptosis, and activation of Nrf2 and genes governed by it despite using a lower concentration of the compound. Furthermore, it was concluded that Nrf2 activation occurred through Re stimulation of the PI3K/Akt/Nrf2 and p-ERK/Nrf2 pathways [85]. On the other hand, in vivo assays have been performed with mice with memory deficits caused by chronic restraint stress. Treatment with different doses of Re increased memory and prevented the loss of neurons in the hippocampus. As the in vitro studies showed, an increase in the amount of Nrf2, HO-1, SOD, GSH, and CAT was evident, resulting in an alleviation of OS [103].

Ginsenoside Rg1: Ginsenoside Rg1 was shown to decrease prooxidant activity in corticosterone-stimulated PC12 cells in a concentration-dependent manner. Rg1 also increased the expression of Nrf2 and HO-1 but decreased levels of GAS5 (Growth Arrest Specific 5), a lncRNA (long noncoding RNA) implicated in physiological alterations. These findings were confirmed by using shRNA (short hairpin RNA) to repress GAS5 expression, demonstrating that GAS5 inhibited Nrf2 expression through EZH2 (histone lysine methyltransferase 2). This same effect of Rg1 on Nrf2 and GAS5 was observed in mice subjected to chronic restraint stress, resulting in improved depressive behavior. Thus, Rg1 exerts a protective effect by downregulating GAS5, which increases Nrf2 levels, leading to an antioxidant response [86]. 

In PC12 cells subjected to nutritional stress, treatment with Rg1 (1 μM) was found to have a protective effect that depends on the Nrf2/HO-1 pathway. The results showed that a lack of glucose and oxygen led to an increase in the expression of miR-144, a miRNA that binds to the 3′-untranslated region of the Nrf2 gene, thereby preventing its expression. Furthermore, in an in vivo model of ischemia/reperfusion injury, where miR-144 expression was inhibited, treatment with Rg1 (20 mg/kg) was ineffective. Thus, suppression of miR-144 mimicked the effects produced by Rg1. In conclusion, Rg1 inhibits the action of miR-144 that downregulates Nrf2 expression and, as a result, genes with antioxidant activity are expressed, which help mitigate the damaging effect of nutrient deficiency in nervous tissue [87].

Ginsenoside Rb1: Chronic social defeat stress (CSDS) has been shown to induce oxidative damage and inflammation in the mouse hippocampus. Ginsenoside Rb1 was found to reverse the effects of CSDS by activating Nrf2 and genes such as SOD and CAT, as well as reducing the activity of NLRP3 (NLR family pyrin domain-containing 3) inflammasome and proinflammatory cytokines. The regulation of these pathways was observed to be governed upstream by SIRT1, the levels of which increased with Rb1 treatment [104]. The neuroprotective effect of Rb1 through activation of the Nrf2/HO-1 pathway was also documented in models of pentylenetetrazol-induced brain injury in SD rats and Mg^2+^-free-induced neuron injury. In addition, a decrease in apoptosis, inflammation, and lipid peroxidation levels was observed [105].

Ginsenoside Rd: The protective effect of ginsenoside Rd against ischemic/reperfusion injury was evaluated in both in vivo (using tMCAO mice) and in vitro (using primary cortical neurons deprived of glucose and oxygen) models. The study found that Rd protected against pyroptosis induced by ischemic/reperfusion injury by upregulating miR-139-5p, an inhibitor of FoxO1 (Forkhead box protein O1). The activation of miR-139-5p resulted in the inhibition of FoxO1, leading to the increased expression of Keap-1, which in turn inhibited Nrf2. Rd then activated Nrf2, triggering the activation of genes that neutralized ROS and decreased NLRP3 inflammasome activation, ultimately reducing neuronal damage [106].

Compound K: The ginsenosides of the protopanaxadiol saponin group can be converted by the gut microbiome into compound K (CK). CK is then absorbed by the organism and has an antioxidant effect. The impact of CK on the Nrf2/HO-1 pathway was studied in mice with memory impairment produced by scopolamine hydrobromide (SCOP). Treatment with SCOP alone resulted in memory impairment, increased apoptosis, and decreased Aβ-peptide clearance capacity in ICR mice. However, when treatment was combined with CK (20 or 40 mg/kg), the opposite effects were observed, along with an upregulation of Nrf2 and HO-1 levels and a decrease in Keap-1. These results suggest that the neuroprotective effects of CK may be mediated by Nrf2 activation [107].

### 4.4. Carotenoids

Carotenoids are a group of terpenoid compounds comprising eight isoprene units. They are brightly colored pigments in hues of red, orange, and yellow that are found in vegetables, plants, algae, and fungi. They have been shown to have numerous health benefits, including antioxidant, anti-inflammatory, antimicrobial, and UV (ultraviolet) radiation protection properties [120].

*Euglena gracilis* is a flagellated microalga known for its high concentration of carotenoids. In an in vitro study, the anti-inflammatory effects of the carotenoid-rich acetone extract (AE) of *E.gracilis* were evaluated using microglia obtained from SD rats. The composition of the extract was determined by HPLC (high-performance liquid chromatography) and found to contain neoxanthin (17%), diadinoxanthin (45%), canthaxanthin (10%), zeaxanthin (14%), and β-carotene (8%). AE (100 ng/mL) showed anti-inflammatory effects on LPS-stimulated microglia through an inhibition of NF-κB, resulting in decreased expression of proinflammatory cytokines (tumoral necrosis factor-α and IL-1β). These effects were also observed to be mediated by an increase in Nrf2 and HO-1 mRNA levels. Moreover, pharmacological inhibition of Nrf2 (using ML385) did not significantly affect the anti-inflammatory activity of the extract, suggesting that the NF-κB and Nrf2 pathways function independently in this instance [88].

#### 4.4.1. Astaxanthin

One of the carotenoids whose studies have increased notably is astaxanthin (ATX); it is a β-carotenoid extracted mainly from the green algae *Haematococcus pluvialis*, which has been shown to have a great capacity to eliminate ROS both internally and externally in the cell membrane [121,122].

The in vitro studies on the antioxidant activity of ATX in SH-SY5Y cells showed promising results. Zhang et al. (2020) employed this cell line that had previously undergone OGD to determine antioxidant activity. It was observed that ATX pretreatment not only significantly improved cell viability but also reduced ROS and membrane oxidation levels and increased SOD activity. On the other hand, ATX also improved mitochondrial membrane potential and stimulated the expression of HO-1 and Nrf2 proteins. Additionally, the increased expression of Nrf2 and HO-1 was reversed when co-treated with a PI3K inhibitor, but a GSK-3 inhibitor promoted Nrf2 translocation. These results suggest that ATX protects against OGD through the PI3K/GSK-3/Nrf2 pathway [89]. Continuing with the same cell line, the protective effect of ATX at the mitochondrial level and antioxidant capacity in cells subjected to glutamate-mediated excitotoxicity was studied. At the level of antioxidant activity, pretreatment with ATX at a dose of 20 µM decreased the levels of MDA, protein carbonylation, and the 8-oxo-2′-deoxyguanosine marker. In the mitochondria, ATX decreased free radical production. However, siRNA silencing of Nrf2 was observed to block the effect of ATX on HO-1 activity [90]. Later, they treated the SH-SY5Y cells with H_2_O_2_ as a stressor to produce redox impairment and mitochondrial dysfunction. Upon pretreatment with ATX, they observed similar results to those obtained above. In contrast, ATX increased HO-1 activity and Nrf2 activity via a mechanism associated with the PI3K/Akt signaling pathway [91].

In animal models such as SD rats, pretreatment with ATX has been shown to ameliorate neurological dysfunction induced by acute cerebral infarction. Regarding antioxidant enzymes, ATX improved the levels of CAT, SOD, and GPx and reduced lipid peroxidation. After acute cerebral infarction, Western blot analysis revealed that ATX enhanced the expression of HO-1 and nuclear Nrf2 and decreased the expression of cytosolic Nrf-2 [108]. In the same way, in another study using C57BL/6 mice induced with a moderate controlled TBI, ATX improved neuronal function. Immunofluorescence staining showed that ATX is also able to promote the nuclear import of Nrf2. ATX, compared to the control group, increased the levels of HO-1 and Nrf2 proteins [109]. The protective effects of ATX against La_2_O_3_-nanoparticle-induced neurotoxicity were evaluated in Kunming mice. The levels of various antioxidant proteins such as SOD, GPx, and GSH were measured and found to be elevated in the ATX-pretreated group. Additionally, ATX restored the levels of PI3K p110, PI3K p85, p-AKT, Nrf-2, HO-1, NQO1, and GCLM proteins compared to the control group [110]. An evaluation of the antioxidant activity of ATX extracted from the microalgae *H. pluvialis* was performed in a Wistar rat model of non-arteritic anterior ischemic optic neuropathy (NAION). The results demonstrated that both pre- and post-injury treatment with ATX increased the levels of SOD, Nrf2, and p62 compared to the control rats [92].

#### 4.4.2. Other Carotenoids

Another carotenoid present in algae is fucoxanthin (FCX). In experiments carried out in a primary culture of cortical neurons subjected to OGD, treatment with increasing concentrations of FCX (5–20 μM) showed a neutralization of ROS, a decrease in apoptosis, increased SOD activity, and an increase in Nrf2 activation and consequent HO-1 expression. Moreover, the inhibition of Nrf2 expression by siRNA reversed the decrease in apoptosis produced by FCX. These results showed that FCX exerted its protective activity through the Nrf2/HO-1 pathway [93]. FCX also exhibited Nrf2-mediated antiapoptotic and antioxidant activity in PC12 cells stressed with 6-ODHA. Complementarily, it was determined that the mechanism of action of this algal carotenoid consists of the interaction through two hydrogen bonds with residues Arg415 and Tyr525 of the protein pocket of Keap-1, which prevents its association with Nrf2, enhancing the activity of the factor and the antioxidative enzymes it regulates [94].

Lycopene is a pigment found in foods such as tomato, apricot, and watermelon. Its antioxidant properties have been demonstrated in in vitro studies in BV2 cells. Treatment with lycopene (50 μM) prior to stimulation with LPS reduced OS and amyloidogenesis, decreased ROS levels, preserved mitochondrial membrane potential, and increased the expression of Nrf2 and genes regulated by it (HO-1 and NQO-1) [95]. In green carp (*Ctenopharyngodon idella*), sulfamethoxazole (SMZ) caused neurotoxicity, damaging the blood–brain barrier and increased OS, inducing inflammation and causing apoptosis. Coadministration of lycopene (10 mg/kg) ameliorated the toxic effects of SMZ. In this case, it was observed that SMZ greatly elevated NF-κB and Nrf2 levels, which was interpreted as a defense mechanism against the drug, whereas lycopene with SMZ produced a reduction in the activation of both pathways, suggesting that this carotenoid was able to neutralize the damage and restore homeostatic conditions [111]. In an in vivo model of hypoxic–ischemic brain injury with neonatal SD rats, treatment with lycopene (10 mg/kg) showed a decrease in brain infarction, inflammation, and apoptosis, as well as an improvement in memory and learning. The mechanism involved in the protective action was determined to be the activation of Nrf2, which resulted in the inhibition of NF-κB and, consequently, a lowering of inflammation and neuronal damage [112]. The potential of lycopene as an antioxidant therapy against aluminum-induced neurotoxicity in the hippocampus was also studied. In Wistar rats, daily feeding of AlCl_3_ caused hippocampal injury, memory impairment, oxidative damage, inflammation, and apoptosis. Co-treatment with lycopene (4 mg/kg) reduced the toxicity of aluminum by inducing Nrf2 translocation to the nucleus and expression of antioxidative enzymes (HO-1, NQO-1, GCLC, and SOD-1) [113].

Lutein and zeaxanthin are two isomeric carotenoids present in vegetables, and they are the most abundant in human brain tissue. They are also known to have protective activity on neurons. In murine models of TBI, the combined administration of both carotenoids (20 mg/kg) produced a decrease in infarct volume and edema. On the other hand, it was determined that the treatment produced an increase in Nrf2 activation, which neutralized OS and inhibited NF-κB, which attenuated inflammation [114]. In another study in Wistar rats, the inhibition of NF-κB but not Nrf2 or HO-1 by lutein/zeaxanthin (100 mg/kg) was observed. However, the combination of carotenoids with physical exercise led to a greater activation of the Nrf2/HO-1 pathway than exercise without treatment [115].

Torularhodin (TOR) is a fungal carotenoid that possesses anticancer and antimicrobial activity and, above all, has been shown to have a strong antioxidant activity, which is related to the presence of 13 double bonds in its chemical structure [123]. The neuroprotective capacity of TOR has been evaluated at different concentrations in H_2_O_2_-stressed HT22 cells. ROS levels were reduced in those cells that had been treated with TOR isolated and purified from *Sporidiobolus pararoseus*, also achieving the restoration of the mitochondrial membrane potential. Cells that were treated with TOR had increased HO-1 expression levels and nuclear levels of Nrf2 and decreased NF-κB protein levels. In vivo studies using ICR mice have studied the neuroprotective activity of TOR in situations of OS by D-galactose/AlCl_3_. Analysis of antioxidant enzymes showed how treatment with TOR at a concentration of 1.5 mg/kg generated an increase in the levels of SOD, CAT, and GPx compared to the control groups. In the Nrf2 signaling pathway, TOR treatment generated an increase in Nrf2 and HO-1 expression and a decrease in NF-κB expression [96].

β-Carotene is mainly found in higher plants, but also in bacteria, fungi, and algae [124]. Its effect on the Nrf2 pathway has not been well studied, but a recent study in C57BL/6 mice induced with TBI showed its neuroprotective activity. The study used a dose of 30 mg/kg of β-carotene and found an increase in SOD levels, Nrf2 nuclear localization (detected by immunofluorescence staining), and HO-1 and NQO-1 protein levels as well as reduced Keap-1 protein levels [116].

Crocin is a water-soluble carotenoid present in various plant species whose antioxidant activity has been evaluated in several studies [125,126]. Recently, Wang et al. (2022) evaluated the protective capacity of crocin in an in vivo model of C57BL/6 mice in which intracerebral hemorrhage (ICH) was induced. The enzyme levels of SOD and GPx in the crocin-treated groups increased after ICH. Ferroptosis was also inhibited because of this compound; the levels of GPx4, ferritin heavy chain 1 (FTH1), and SLC7A11 were analyzed by Western blot, showing an upregulation of these proteins which are regulated by the Nrf2 pathway. Finally, the study found that crocin treatment led to decreased cytoplasmatic expression but increased nuclear expression of Nrf2 [117].

## 5. Discussion and Future Perspectives

Natural products and plant extracts exhibit diverse and synergistic effects contributing to a therapeutic outcome. The Nrf2/ARE pathway plays a critical role in mitigating OS, but there are alternative pathways capable of neutralizing ROS. These include the ferroptotic pathway, apoptotic pathway, FoxO pathway, NF-κB pathway, and MAPK pathway. The pathways may intersect, and components of the cascade may participate in multiple pathways simultaneously [127]. Many of the phytochemicals reviewed in this study act as modulators of various signaling cascades and target components involved in cross-pathway interactions. The pathways often converge on Nrf2, a transcription factor that regulates the expression of genes involved in reducing OS. As a result, these natural products can regulate multiple pathways with crosstalk, enhancing the cell’s capacity to combat OS. 

This review discusses various methods of inhibiting Nrf2, including siRNA, Nrf2 knockout murine models, and pharmacological inhibitors (trigonelline [31,56] and ML385 [39,88]), demonstrating that the neuroprotective effects of many natural compounds rely on Nrf2, regardless of the initial receptor targeted. Nrf2 is therefore established as a key molecular component in ROS elimination.

The tradition of utilizing natural resources to uncover active ingredients that can promote human health dates back to traditional Chinese medicine, which heavily relies on plant-based remedies. Today, medicinal plants are a significant source of compounds with clinical applications, such as capsaicin and paclitaxel. Many compounds are still undergoing preliminary research to fully understand their pharmacological properties, including quercetin, curcumin, and EGCG [128]. Plant secondary metabolites, despite their significance, are present in low quantities in raw materials, thus necessitating isolation through techniques such as solvent extraction or distillation. However, advancements in extraction methods, such as ultrasound, microwave, or supercritical fluid extraction, have been made and have led to improved yields and purity of extracted products [129]. The advancement in extraction techniques makes it feasible to identify a greater number of metabolites from under-explored sources, thereby facilitating progress in the discovery of new drugs, especially antioxidants, which have the potential to prevent cardiovascular, metabolic, and neurodegenerative diseases.

In this review, various compounds have been discussed, some of which have undergone clinical trials that have demonstrated their effectiveness in treating certain neurological disorders, such as anxiety, migraine, memory loss, and age-related cognitive decline. Compounds such as curcumin [130,131,132,133], EGCG [134], carotenoids [135], ginkgo leaf extract [136], and ginseng [137] have been studied in this regard. The potential improvement in patients’ cognitive abilities might be attributed to the molecular mechanisms outlined in this review; however, further in vitro and in vivo studies, as well as additional clinical trials, are necessary to thoroughly understand the efficacy and safety of these products. Such understanding is crucial for their effective utilization in combating NDs. 

Furthermore, active compounds that act on the central nervous system must traverse the blood–brain barrier (BBB) to impart their therapeutic effects, making the permeability of this cellular barrier a crucial factor in the development of drugs aimed at this region. The BBB permeability of natural products has been the subject of numerous studies due to their promise as treatments for various neurological disorders. In 2010, Fang et al. evaluated the ability of ginkgolide B to penetrate the BBB in in vitro and in vivo models of cerebral ischemia [138]. The presence of this compound in neural tissue is attributed to its cognitive-enhancing effects in the elderly population. Recently, Shimazu et al. conducted an analysis of the BBB permeability of several phenolic compounds and found that EGC had high permeability, while quercetin and kaempferol had intermediate permeability, and curcumin and anthocyanins displayed low permeability [139]. The permeability of terpenoids has also been investigated, as demonstrated in a study by Sánchez-Martínez et al. who utilized terpenoid compounds (monoterpenes and sesquiterpenes) derived from orange waste to assess their neuroprotective properties. The tested compounds exhibited high permeability through the BBB in vitro, a result anticipated due to their lipophilic nature [140]. Currently, efforts are underway to identify new plant sources with potential neuroprotective properties. This includes trials to assess their efficacy by evaluating their ability to penetrate the BBB [141]. Moreover, efforts are underway to improve the delivery of natural products with low BBB permeability, such as curcumin, through the use of nanoparticles [142] or the direct synthesis of chemically modified analogs with improved permeability [143].

As such, there is still much room for exploration in the pharmacology of neuroprotective natural products. This includes identifying new sources of active ingredients, optimizing their extraction processes, and conducting further research on their effects on humans and their ability to penetrate the central nervous system and reach their molecular targets.

## 6. Conclusions

Natural products hold potential as treatments for nervous system diseases caused by oxidative stress, such as AD or PD. In many cases, the protective effects are mediated through activation of the Nrf2 pathway, a transcription factor that upregulates genes encoding enzymes that scavenge ROS. Quercetin and curcumin are among the most extensively researched natural products due to their antioxidant properties and have been shown to activate Nrf2. Other less studied phenolic compounds also possess this activity. Terpenoids, ranging from monoterpenes to carotenoids, are phytochemicals that have demonstrated their ability to modulate Nrf2 as a mechanism to combat oxidative damage. Of particular interest are ginsenosides and ginkgolides, found in plants significant in traditional Chinese medicine. Given the multitude of Nrf2-modulating secondary metabolites, it is crucial to further our understanding of these compounds in order to develop drugs that effectively mitigate oxidative stress and treat disabling diseases affecting the nervous system.

## Figures and Tables

**Table 1:** Modulation of phenolic compounds on Nrf2 pathway in in vitro models. The increase in protein or RNAm levels are represented with (↑) and decrease is represented with (↓).

Treatment	Experimental Model	Effects on Nrf2 Pathway	Ref.
Metanolic extract of *Dendropanax morbifera* leaves, quercetin, or isoquercetin	Glutamate-induced oxidative stress (OS) in HT22 cells.	Three treatments ↑ Nrf2 and HO-1 protein levels.	[29]
Quercetin	7-ketocholecterol-induced oxiapoptophagy in N2a cells.	↑ Nrf2 and SOD-1 mRNA levels. ↑ Nrf2, SOD-1, SOD-2, CAT, and GPx protein levels. ↑ SOD and GPx activities.	[30]
	Corticosterone-induced cytotoxicity in primary cortical neurons.	Protective effect of quercetin is not mediated by Nrf2.	[31]
	ΔK280 TauRD-DsRed SH-SY5Y cells.	↑ Nrf2 protein levels (maybe mediated by TRKB/Akt pathway).	[32]
	High glucose concentration in SH-SY5Y cells.	↑ Nrf2 and p-NRF2 protein levels (maybe mediated by PKC activation and/or GSK-3β inhibition). ↑ Nrf2 nuclear levels.	[33]
	Aβ_25-35_-induced cytotoxicity in PC12 cells.	↑ HO-1 mRNA and protein levels. ↓ Nrf2 and Sirtuin-1 (SIRT1) mRNA and protein levels. ↑ SOD, CAT, and GPx activities.	[34]
	Acrylamide-induced cytotoxicity in C6 cells.	↑ Nrf2 nuclear levels.	[35]
Curcumin	Immortalized mouse cortical neuronal cells.	↑ Nrf2, HO-1, NQO-1, and GST mRNA and protein levels (maybe mediated by PKCδ/p62/Nrf2 pathway) ↑ Nrf2 nuclear levels. ↑ ARE activity.	[36]
	Ethanol-induced OS in HT22 cells.	↑ Nrf2 and HO-1 protein levels.	[37]
	Methylmercury-induced cytotoxicity in primary rat astrocytes.	↑ Nrf2, HO-1, NQO-1 and GSH protein levels (independently of PKCδ). ↑ Nrf2 nuclear levels. ↑ CAT activity.	[38]
	Heme-induced OS in HAPI cells and primary rat cortical microglia.	↑ Nrf2, HO-1, NQO-1, and GPx4 mRNA and protein levels.	[39]
	Pam3CSK4-stimulated BV2 cells	↑ Nrf2 and HO-1 protein levels. ↑ Nrf2 nuclear levels.	[40]
Hydroxytyrosol	6-OHDA-induced cytotoxicity in SH-SY5Y cells.	No changes in Nrf2 protein levels. No changes in HO-1 mRNA and protein levels.	[41]
Mixture of oleuropein, *p-*coumaric acid, and tyrosol.	H_2_O_2_-induced oxidative damage in SK-N-SH cells.	↓ Nrf2 protein levels.	[42]
Anthocyanins purified from methanolic extract from Korean black bean	AβO-induced cytotoxicity in HT22 cells.	↑ Nrf2, p-PI3K, p-Akt, p-GSK-3β, HO-1, and GCLM protein levels (reversed by Nrf2 and p-PI3K inhibitors).	[43]
Cyanidin-3-glucoside	Glutamate-induced cytotoxicity in HT22 cells.	↑ Nrf2 and ERK protein levels. ↑ SOD-1, SOD-2, CAT, and GPx mRNA levels.	[44]
Proanthocyanidins	Cypermethrin-induced OS in mouse cortical neurons.	↓ Nrf2, HO-1, and NQO-1 mRNA and protein levels. ↑ Keap-1 protein levels.	[45]
Kaempferol	OGD/R-induced ferroptosis in primary mouse cortical neurons.	↑ Nrf2, SLC7A11, and GPx4 protein levels. ↑ GSH and SOD activities.	[46]
Tiliroside	BV2 and HT22 cells.	↑ Nrf2, HO-1, NQO-1, and SIRT1 protein levels in BV2 cells. ↑ Nrf2, HO-1, and NQO-1 protein levels in HT22 cells.	[47]
Engeletin	Aβ_1-42_-induced OS and neuroinflammation in BV2 cells.	↑ Nrf2 protein levels. ↑ Nrf2 nuclear levels. ↓ Keap1 protein levels. ↑ SOD and GPx activities.	[48]
Icariin	6-OHDA-induced neuroinflammation in mouse neuron–microglia co-culture.	Inhibition of Nrf2 reversed protective effect of icariin in microglia cells, but not in neurons.	[49]
Isoliquiritigenin	AβO-induced neuroinflammation in BV2 cells.	↑ Nrf2, HO-1, and NQO-1 mRNA and protein levels.	[50]
Pinocembrin-7-methyleter	6-OHDA-induced neurotoxicity in SH-SY5Y cells.	↑ Nrf2 nuclear levels (maybe mediated by ERK and Akt). ↑ SOD and GPx activities. ↑HO-1 and NQO-1 mRNA and protein levels.	[51]
Garlic acid (GA), epigallocatechin (EGC), epicatechin-3-gallate (EGG), epigallocatechin-3-gallate (EGCG), theaflavin (TF), and tannic acid (TA)	HEK293T and SH-SY5Y cells.	GA, EGG, and TA ↑ ARE activity in HEK293T. GA, EGG, and TA ↑ HO-1 protein levels in SH-SY5Y cells. EGC, EGCG, and TF did not have these effects.	[52]
TF from ethanolic extract of black tea	OGD/R-induced OS in PC12 cells.	↑ HO-1 protein levels. ↑ Nrf2 nuclear levels. ↑ SOD activity.	[53]
EGCG	CoCl_2_-induced hypoxia in BV2 cells.	↑ HO-1 protein levels. ↑ Nrf2 nuclear levels.	[54]

**Table 2:** Modulation of phenolic compounds on Nrf2 pathway in in vivo models. The increase in protein or RNAm levels are represented with (↑) and decrease is represented with (↓).

Treatment	Experimental Model	Effects on Nrf2 Pathway	Ref.
Quercetin	Aβ_42_ supplied intracranially in SD rats.	↑ Nrf2, HO-1, SOD, CAT, and GSH protein levels.	[55]
	Streptozotocin supplied intracerebroventricularly in Wistar rats.	↑ HO-1 protein levels (maybe mediated by α7nAChR/Nrf2 pathway).	[56]
	Energy-drink-induced neurotoxicity in Wistar rats.	↑ Nrf2 and HO-1 mRNA and protein levels.	[57]
	Traumatic brain injury in SD rats.	↑ Nrf2 nuclear and cytoplasmatic protein levels. ↑ HO-1 protein levels. ↑ SOD, CAT, and GPx activities.	[58]
	Chronic unpredictable mild stress in Kunming mice.	↑ Nrf2, HO-1, p-PI3K, and p-Akt protein levels. ↑ SOD and glutathione-S-transferase (GST) activities.	[59]
	t-MCAO in Wistar rats.	↑ Nrf2, HO-1, and SIRT1 protein levels.	[60]
Curcumin	Traumatic brain injury in ICR mice.	↑ Nrf2 nuclear levels. ↑ HO-1 and NQO-1 protein levels. ↑ SOD and GPx activities.	[61]
	Traumatic brain injury in Nrf2-knockout mice and WT.	↑ Nrf2, HO-1, and NQO-1 mRNA and protein levels in WT mice. ↓ HO-1 and NQO-1 mRNA and protein levels in Nrf2-knockout mice	[62]
	Chronic unpredictable mild stress in SD rats.	↑ Nrf2, HO-1, and NQO-1 mRNA levels. ↑ Nrf2 nuclear levels.	[63]
	Quinolinic-acid-induced neurotoxicity in Wistar rats.	↑ Nrf2 protein levels (maybe mediated by BDNF/TRKB/ERK pathway). ↑ CAT, GSH, SOD, and GPx activities.	[64]
	Arsenic-trioxide-induced neurotoxicity in Sanshui white ducks.	↑ Nrf2, SOD-1, HO-1, CAT, GPx1, and thioredoxin mRNA and protein levels. ↓Keap1 mRNA and protein levels.	[65]
Olive dry extract enriched in hydroxytyrosol 20%	*Caenorhabditis elegans* wild-type and *C. elegans* mutants.	↑ SKIN-1 (same function that Nrf2).	[66]
Anthocyanins purified from methanolic extract from Korean black bean	Double-mutant APP/PS1 mice as AD model.	↑ Nrf2 nuclear levels (maybe mediated by PI3K/Akt/GSK-3β pathway). ↑ HO-1 and GCLM protein levels.	[43]
Proanthocyanidins	Cypermethrin-induced OS in mouse cortical neurons.	↓ Nrf2, HO-1, and NQO-1 mRNA and protein levels. ↑ Keap-1 protein levels.	[45]
Icariin	6-OHDA-induced neuroinflammation in WT and Nrf2 knockout mice.	↑ Nrf2, HO-1, and NQO-1 mRNA and protein levels in WT mice. ↑ Nrf2 nuclear levels in WT mice. Nrf2 knockout mice did not have these effects.	[49]
Hesperetin	Aβ_1-42_-induced neurotoxicity in brain mice.	↑ Nrf2 and HO-1 protein level.	[67]
Ethanolic extract of *Abelmoschus esculentus* flowers	TCIRI-induced OS in Kunming mice.	↑ Nrf2, HO-1, and NQO-1 protein levels.	[68]
EGCG	CCH-induced cognitive impairments in SD rats.	↑ Nrf2, HO-1, PI3K, p-Akt, and SOD protein levels. ↑ HO-1 activity.	[69]

**Table 3:** Modulation of terpenoids on Nrf2 pathway in in vitro models. The increase in protein or RNAm levels are represented with (↑) and decrease is represented with (↓).

Treatment	Experimental Model	Effects on Nrf2 Pathway	Ref.
Aucubin	H_2_O_2_-induced OS in SH-SY5Y cells.	↑ Nrf2, HO-1, and NQO-1 protein levels. ↑ SOD, CAT, GSH, and GPx activities. ↓ Keap-1 protein levels.	[76]
	H_2_O_2_-induced OS in primary cortical neurons.	↑ HO-1 and NQO-1 protein levels. ↑ Nrf2 nuclear levels.	[77]
Dihydroactinidiolide	SH-SY5Y cells intoxicated with glutamate, colchicine, sodium dithionite, and amyloid β.	↑ Nrf2 and HO-1 mRNA levels.	[78]
Paeoniflorin	Glutamate-induced excitotoxicity in SH-SY5Y cells.	↑ Nrf2, HO-1, and NQO-1 protein levels. ↓ Keap-1 protein levels. ↑ SOD and GSH activity.	[79]
6’-*O*-galloylpaeoniflorin	OGD-induced OS in PC12 cells.	↑ Nrf2 nuclear levels. ↑ p-Akt protein levels. ↑ SOD activity.	[80]
Ginkgolides A, B, and K	OGD/R-induced OS in SH-SY5Y cells.	GB ↑ SOD activity. GB ↑ p-Nrf2, HO-1, NQO-1, and p-Akt protein levels. GA ↑ Nrf2 and NQO-1 protein levels. GK ↑ HO-1 protein levels.	[81]
Ginkgolides A, B, and C	OGD/R-induced OS in PC12 cells.	Three ginkgolides ↑ Nrf2, p-Akt, and HO-1 protein levels.	[82]
Diterpene ginkgolides meglumine injection	OGD/R-induced OS in PC12 cells.	↑ Nrf2 and p-Akt protein levels.	[82]
Ginkgolide K	OGD/R-induced OS in primary cortical astrocytes culture.	↑ Nrf2 and HO-1 protein levels.	[83]
Ginsenoside Re	Aβ-induced neurotoxicity in SH-SY5Y cells.	↑ Nrf2 nuclear levels. ↑ HO-1, NQO-1, and GCLC mRNA levels. ↑ GSH protein levels. ↑ SOD and GPx activities.	[84]
	Rotenone-induced PD model in SH-SY5Y cells.	↑ Nrf2 nuclear levels (maybe mediated by PI3K/Akt or ERK pathway). ↑ HO-1, NQO-1, and GCLC protein levels.	[85]
Ginsenoside Rg1	Corticosterone-induced OS in PC12 cells.	↑ Nrf2 and HO-1 protein levels.	[86]
	OGD/R-induced OS in PC12 cells.	↑ Nrf2 nuclear levels (maybe mediated by inhibition of mi-R144). ↑ HO-1, NQO-1, GCLC. and GCLM mRNA and protein levels. ↑ ARE activity. ↓ Keap1 protein levels.	[87]
Carotenoid-rich acetone extract of *Euglena gracilis*	LPS-stimulated cortical microglia.	↑ Νrf2 and HO-1 mRNA levels.	[88]
Astaxanthin	OGD-induced OS in SH-SY5Y cells.	↑ Nrf2 nuclear level (maybe mediated by PI3K/GSK-3 pathway). ↑ HO-1 protein level. ↑ SOD activity.	[89]
	Glutamate-induced excitotoxicity in SH-SY5Y cells.	↑ Nrf2 and HO-1 activities.	[90]
	H_2_O_2_-induced OS in SH-SY5Y cells.	↑ Nrf2 and HO-1 activities (maybe mediated by PI3K/Akt pathway).	[91]
Astaxanthin extracted from *Haematococcus pluvialis*	NAION in Wistar rats.	↑ Nrf2 protein level. ↑ SOD activity.	[92]
Fucoxanthin	OGD/R-induced OS in neuron culture.	↑ HO-1 protein levels. ↑ Νrf2 nuclear levels.	[93]
	6-ODHA-induced neurotoxicity in PC12 cells.	Fucoxanthin prevents physical association between Nrf2 and Keap-1.	[94]
Lycopene	LPS-induced neuroinflammation and OS in BV12 cells.	↑ Nrf2 nuclear levels. ↑ HO-1 and NQO-1 protein levels. ↑ SOD, CAT, and GSH activities.	[95]
Torularhodin extracted from *Sporidiobolus pararoseus*	H_2_O_2_-induced OS in HT22 cells.	↑ Nrf2 nuclear levels. ↑ HO-1 protein levels.	[96]

**Table 4:** Modulation of terpenoids on Nrf2 pathway in in vivo models. The increase in protein or RNAm levels are represented with (↑) and decrease is represented with (↓).

Treatment	Experimental Model	Effects on Nrf2 Pathway	Ref.
Aucubin	Traumatic brain injury in C57BL/6 mice.	↑ HO-1 and NQO-1 protein levels. ↑ SOD, GSH, and GPx activities. ↑ Nrf2 nuclear levels.	[77]
6’-*O*-galloylpaeoniflorin	Wistar rat ischemia–reperfusion injury model.	↑ Nrf2 nuclear levels. ↑HO-1 protein levels. ↑ SOD, GSH, and GPx activity.	[80]
Carveol	Pentylenetetrazole-induced epilepsy in SD rats.	↑ Nrf2 and HO-1 mRNA levels. ↑ SOD, CAT, and GST activities.	[97]
	LPS-induced depression in SD rats.	↑ Nrf2 and HO-1 mRNA levels in cortex and hippocampus. ↑ CAT, GSH, and GST activities.	[98]
Catalpol	SD rat depression model.	↑ Nrf2, HO-1, p-PI3K, and p-Akt mRNA and protein levels.	[99]
	Mice depression model.	↑ Nrf2 nuclear levels. ↑ HO-1, p-PI3K, and p-Akt protein levels. ↑ SOD, GSH, GPx, and GST activities.	[100]
	Wistar rat epilepsy model.	↑ Nrf2 protein levels. ↓ Keap-1 mRNA levels. ↑ ARE expression. ↑ SOD activity.	[101]
Ginkgolide K	Cuprizone-induced demyelination in C57BL/6 mice.	↑ Nrf2 and HO-1 protein levels.	[83]
*Xingxiong* injection that has *Ginkgo biloba* leaf extract	SD rat ischemia–reperfusion injury model.	↑ Nrf2, HO-1, and p-Akt protein levels.	[102]
Ginkgolide B	tMCAO-induced OS in SD rats.	↑ Nrf2, p-Nrf2, p-Akt, HO-1, NQO-1, and SOD protein levels.	[81]
Diterpene ginkgolides meglumine injection	SD rats ischemia–reperfusion injury model.	↑ Nrf2, p-Akt, and HO-1 protein levels.	[82]
Ginsenoside Re	Chronic restraint stress in C57BL/6 mice.	↑ Nrf2 and HO-1 protein levels. ↑ SOD, CAT, and GSH activities.	[103]
Ginsenoside Rg1	Chronic restraint stress in SD rats.	↑ Nrf2 protein levels (maybe mediated by inhibition of GAS5).	[86]
	tMCAO-induced ischemia/reperfusion injury in SD rats.	↑ Nrf2 nuclear levels (maybe mediated by inhibition of mi-R144). ↑ HO-1, NQO-1, GCLC, and GCLM mRNA and protein levels.	[87]
Ginsenoside Rb1	Chronic Social Defeat Stress in C57BL/6 mice.	↑ Nrf2 and HO-1 protein levels (maybe mediated by SIRT1). ↑ SOD and CAT activities.	[104]
	Pentylenetetrazol-induced brain injury in SD rat.	↑ Nrf2 and HO-1 protein levels.	[105]
Ginsenoside Rd	tMCAO-induced ischemia/reperfusion injury in C57BL/6 mice.	↑ Nrf2 nuclear levels. ↑ HO-1 and NQO-1 protein levels. ↓ Keap1 mRNA and protein levels (maybe mediated by upregulation of miR-139-5p).	[106]
Compound K	SCOP-induced AD model in ICR mice.	↑ Nrf2 and HO-1 protein levels. ↓ Keap1 protein levels. ↑ SOD and GSH activities.	[107]
Astaxanthin	Acute cerebral infarction in SD rats.	↑ Nrf2 nuclear level. ↑ HO-1 protein level.	[108]
	Traumatic brain injury in C57BL/6 mice.	↑ Nrf2 nuclear level. ↑ Nrf2 and HO-1 mRNA levels. ↑ Nrf2, HO-1, NQO-1, and SOD protein levels.	[109]
	La_2_O_3_-nanoparticles-induced neurotoxicity in Kunming mice.	↑ Nrf2, HO-1, NQO-1, GCLM, PI3K, and p-AKT protein levels. ↑ SOD, GPx, and GSH activities.	[110]
Astaxanthin extracted from *Haematococcus pluvialis*	NAION in Wistar rats.	↑ Nrf2 protein level. ↑ SOD activity.	[92]
Lycopene	Sulfamethoxazole-induced neurotoxicity in *Ctenopharyngodon ιdella.*	↓ Nrf2 and NQO-1 mRNA and protein levels. ↓ HO-1 protein levels. ↑ Keap-1 protein levels.	[111]
	Hypoxic–ischemic injury model in SD rats.	↑ Nrf2 nuclear levels. ↑ HO-1 protein levels.	[112]
	AlCl_3_-induced OS in hippocampus of Wistar rats.	↑ Nrf2 nuclear levels. ↑ HO-1, NQO-1, GCLC, and SOD1 mRNA levels.	[113]
Lutein/zeaxanthin	Traumatic brain injury in C57BL/6.	↑ Nrf2 protein level.	[114]
	Exercised Wistar rats.	Lutein/zeaxanthin combined with physical exercise produced more ↑ SOD and GPx activities and ↑ HO-1 and Nrf2 protein levels than only physical exercise in cerebral cortex.	[115]
Torularhodin extracted from *Sporidiobolus pararoseus*	D-galactose/AlCl_3_-induced OS in ICR mice.	↑ Nrf2 and HO-1 protein levels. ↑ SOD, CAT, and GPx activities.	[96]
β-Carotene	Traumatic brain injury in C57BL/6 mice.	↑ Nrf2 nuclear levels. ↑ HO-1 and NQO-1 mRNA and protein levels. ↓ Keap-1 protein levels.	[116]
Crocin	Intracerebral hemorrhage in C57BL/6 mice.	↑ Nrf2 nuclear levels. ↑ SOD and GPx activities.	[117]

## Data Availability

Data sharing not applicable.
